# Leukoaraiosis is associated with pneumonia after acute ischemic stroke

**DOI:** 10.1186/s12883-017-0830-5

**Published:** 2017-03-16

**Authors:** Ki-Woong Nam, Hyung-Min Kwon, Jae-Sung Lim, Yong-Seok Lee

**Affiliations:** 1 Department of Neurology, Seoul National University College of Medicine, Seoul Metropolitan Government-Seoul National University Boramae Medical Center, 20 Boramae-ro 5-gil, Dongjak-Gu, Seoul, 07061 South Korea; 2 Department of Neurology, Hallym University Sacred Heart Hospital, Anyang, South Korea

**Keywords:** Leukoaraiosis, Cerebral infarction, Pneumonia

## Abstract

**Background:**

Stroke-associated pneumonia (SAP) is common in patients with acute ischemic stroke, and several risk factors have been reported. However, the relationship between underlying leukoaraiosis (LA) and SAP has not been addressed.

**Methods:**

We collected consecutive patients with acute ischemic stroke within 24 h of symptom onset. SAP was defined as the lower respiratory tract infection within the first 7 days after stroke onset, according to the modified Centers for Disease Control and Prevention criteria. LA was graded using the Fazekas scale in both the periventricular and subcortical areas. We evaluated LA burden by summing the grade and dichotomized into mild LA (0–2) or severe LA (3–6). Relationship between LA and SAP was analyzed by binary logistic regression analysis with variables of *P* < 0.05 in univariate analysis.

**Results:**

Three hundred eight consecutive patients were enrolled, and SAP developed in 44 patients (14%). Univariate analysis revealed that SAP correlated with age, initial NIHSS score, atrial fibrillation, impaired consciousness, dysphagia, severe LA and hyperlipidemia. On multivariate analysis, severe LA [adjusted OR (aOR) = 4.41, 95% CI = 2.04–9.55, *P* < 0.001 remained independent predictors of SAP after adjusted confounders.

**Conclusions:**

In this study, LA was an independent predictor of SAP. This observation needs to be confirmed in suitably-designed, prospective studies.

**Electronic supplementary material:**

The online version of this article (doi:10.1186/s12883-017-0830-5) contains supplementary material, which is available to authorized users.

## Background

Pneumonia is a common and significant complication in patients with acute ischemic stroke. The incidence of pneumonia in patients with acute ischemic stroke ranges from 5 to 26% [1, 2]. Stroke-associated pneumonia (SAP) is correlated with poor functional outcome, prolonged hospitalization and high mortality (up to 6-fold) [2, 3]. Thus, rapid assessment of high-risk patients is thought to be needed. Known predictors of SAP include dysphagia, age, male sex, initial stroke severity, non-lacunar stroke type, diabetes, consciousness, atrial fibrillation and acid-suppressive drugs [1, 2, 4, 5].

Leukoaraiosis (LA) is a hyperintense lesion seen in the cerebral white matter of T2-weighted magnetic resonance imaging (MRI) [6], which pathologically correlate with myelin pallor, tissue rarefaction associated with loss of myelin axons, and mild gliosis [7]. LA is frequently found in elderly people, but is especially common and widespread in patients with known vascular risk factors and symptomatic cerebrovascular disease. LA might be important in swallowing and the disruption of cortical-subcortical white matter connections plays an important role in the pathogenesis of dysphagia after stroke [8]. SAP is considered to be the result from the combination of ongoing aspiration and immunological alteration form stroke-induced immunodepression [9, 10]. Thus, the main aim of present study was to investigate that whether larger burden of LA has a positive correlation with SAP in acute ischemic stroke.

## Methods

### Patients

**Fig. 1:**
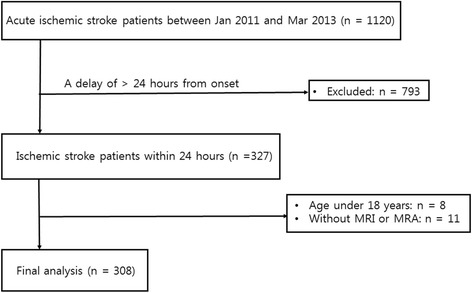
Patient selection flow of the current study

We retrospectively collected a consecutive series of patients with acute ischemic stroke who visited our stroke center between Jan 2011 and Mar 2013 (*n* = 1120). Patients were excluded when they met the following criteria: a delay of >24 h from symptom onset to visiting our center (*n* = 793); age under 18 years (*n* = 8); and patients without brain magnetic resonance imaging (MRI) (*n* = 11). Finally, a total of 308 patients remained for secondary analyses (Fig. 1).

### Clinical assessment

We assessed the following baseline demographic information and risk factors of stroke in all participants: age, sex, hypertension, diabetes, hyperlipidemia [11], atrial fibrillation, and smoking. We also collected initial clinical factors including stroke subtype, stroke location, stroke severity, presence of dysphagia, level of consciousness, and use of thrombolysis therapy. Stroke severity was evaluated using the National Institute of Health Stroke Scale (NIHSS) score on admission and discharge date by trained neurologists. Dysphagia was assessed using a bedside non-instrumented diagnostic test consisting of 3 sequentially performed subtests (semisolid, liquid, and solid textures) [12]. Level of consciousness was dichotomized into normal (NIHSS 1a = 0) and impaired (NIHSS 1a = 1–3). Mechanisms of stroke were classified according to the Trial of Org 10172 in Acute Stroke Treatment classification. The location of the stroke lesions was categorized as either supratentorial or infratentorial. We also collected the information about clinical outcomes including hospitalization duration, discharge NIHSS score, presence of in-hospital mortality, and event of intubation. Patients underwent routine laboratory examination within 24 h from admission including white blood cell count and high sensitivity C-reactive protein levels.

### Radiological evaluation

All participants underwent brain MRI and magnetic resonance angiography within 24 h of the visit with a 3.0-Tesla MR scanner (Achieva 3.0; Philips, Eindohovenm, the Netherlands). The MRI protocol included diffusion-weighted images (DWI) [repetition time (TR)/echo time (TE) = 3000/44 ms], T1-weighted images [TR/TE = 300/10 ms], T2-weighted images [TR/TE = 3000/100 ms], fluid attenuated inversion recovery images [TR/TE = 11,000/120 ms], T2 fast field echo images [TR/TE = 530/16 ms] and three-dimensional time of flight (TOF) MRA images [TR/TE = 24/3.5 ms]. The field of view data in all MRI sequences were 240 × 240 mm. The slice thickness was equally 5.0 mm, excepting 3.0 mm in DWI and 1.2 mm in TOF images. We assessed the severity of the LA using the Fazekas scale in both the periventricular (0–3) and subcortical areas (0–3) [13]. We then summed the grade from the Fazekas scale in both areas and dichotomized this grade into mild LA (sum of grade, 0–2) and severe LA (sum of grade, 3–6) [14]. Two trained neurologists (K.W.N. and J.S.L.) without clinical information graded the severity of LA and the inter-rater reliability coefficient was 0.93. Disagreements were resolved by discussion with a third reviewer (H.M.K.). We also evaluated for the presence of lacunar infarcts and cerebral microbleeds (CMBs). CMBs were divided into lobar CMBs and deep or infratentorial CMBs according to the location of the lesion [15].

### Definition of pneumonia

A patient was diagnosed as SAP if the lower respiratory tract infection, which met the modified Centers for Disease Control (CDC) and Prevention criteria, occurred within the first 7 days after stroke onset (Additional file 1) [16]. The evaluation of presence of SAP was conducted retrospectively by the neurologists (K.W.N. and H.M.K.), who were blinded to other clinical and radiological factors. Additionally, the chest x-ray was evaluated by one of the study neurologist (K.W.N.) and a specialized radiologist (S.W.P.), with an acceptable inter-rater reliability (kappa coefficient, *P* = 0.915). We did not categorize the burden of chest x-ray finding into probable and definite SAP, considering the retrospective study design. During the initial three to four days, we observed all participants closely in our specialized stroke unit. After they were transferred to the general ward, we continued to check for the occurrence of SAP.

### Statistical analysis

All continuous variables were tested for normal distribution, and skewed variables were transformed to log-scale for further statistical analyses. Continuous variables with normal distribution were presented as the mean ± SD, while nonparametric ones were presented using the median value and interquartile range [IQR]. In univariate analyses, we used Student’s *t*-test for normally distributed variables and the Mann-Whitney U-test for nonparametric variables. For categorical values, we used the chi-square test and Fisher’s exact test. In multivariate analyses, we used binary logistic regression to evaluate independent predictors of SAP. Based on the result from the univariate analyses, variables of *P* < 0.05 were selected for the multivariate analysis. We combined prognostic variables of age, atrial fibrillation, dysphagia, male sex, and initial stroke severity into the A2DS2 score to avoid overfitting, considering the small number of SAP events (Additional file 2). The A2DS2 score was dichotomized into low A2DS2 score (0–4) and high A2DS2 score (5–10) [17]. Level of consciousness was excluded due to close correlation with stroke severity (Pearson correlation coefficient, *P* < 0.001). All statistical analyses were conducted using SPSS 20 (IBM SPSS, Chicago, IL, USA). A value of *P* < 0.05 was considered significant.

## Results

We collected a total of 308 patients (mean age = 66 years, the median initial NIHSS score = 4 [2–7]). The mean onset-to-visit time was 6.4 h. SAP occurred in 44 patients (14%).

Baseline characteristics between patients with and without SAP are described in Table 1. In the SAP group, age and the initial NIHSS score were higher compared with the non-SAP group. In addition, atrial fibrillation, impaired consciousness, dysphagia and severe LA were more frequent in the SAP group, while hyperlipidemia was less. The median A2DS2 score was 2 and high A2DS2 score was more frequent in the SAP group.

**Table 1:** Baseline characteristics between with and without stroke associated pneumonia

	Non-SAP (*n* = 264)	SAP (*n* = 44)	*P* value
Age, y	65 [56–74]	71 [66–79]	<0.001
Sex, male (%)	161 (61)	31 (70)	0.230
Hypertension (%)	185 (70)	34 (77)	0.330
Diabetes (%)	83 (31)	17 (39)	0.345
Hyperlipidemia (%)	110 (42)	10 (23)	0.017
Atrial fibrillation (%)	40 (15)	18 (41)	<0.001
Smoking (%)	132 (50)	22 (50)	1.000
Initial total NIHSS [IQR]	3 [1–6]	11 [4–19]	<0.001
Level of consciousness (%)			<0.001
Normal (NIHSS 1a = 0)	251 (95)	28 (64)	
Impaired (NIHSS 1a = 1–3)	13 (5)	16 (36)	
A2DS2 score			<0.001
Low (0–4)	219 (85)	18 (43)	
High (5–10)	38 (15)	24 (57)	
Thrombolysis (%)			0.626
None	237 (90)	39 (89)	
Intravenous	23 (9)	4 (9)	
Intra-arterial	2 (1)	0 (0)	
Both	2 (1)	1 (2)	
Dysphagia (%)	34 (13)	24 (56)	<0.001
Stroke subtype (%)			0.056
Large artery disease	93 (35)	18 (41)	
Cardioembolism	51 (19)	18 (41)	
Small vessel occlusion	92 (35)	3 (7)	
Undetermined	28 (11)	5 (11)	
Stroke location (%)			0.466
Supratentorial	203 (77)	33 (75)	
Infratentorial	54 (20)	8 (18)	
Both	7 (3)	3 (7)	
Lacunar infarcts (%)	73 (30)	10 (28)	0.804
Lobar cerebral microbleeds (%)	42 (17)	6 (17)	0.968
Deep/infratentorial cerebral microbleeds (%)	59 (24)	11 (30)	0.426
Leukoaraiosis (%)			<0.001
Mild (0–2)	190 (75)	15 (38)	
Severe (3–6)	62 (25)	24 (62)	
White blood cell, ×10^3^/μl	7.26 [6.09–9.01]	7.82 [6.67–8.99]	0.149
hs-CRP, mg/dL [IQR]	0.11 [0.05–0.25]	0.15 [0.05–1.36]	0.126

*SAP* stroke-associated pneumonia, *NIHSS* National Institute of Health Stroke Scale, *hs-CRP* high-sensitivity C-reactive protein

According to multivariate analyses, severe LA [adjusted OR (aOR) = 4.41, 95% CI = 2.04–9.55, *P* < 0.001], hyperlipidemia (aOR = 0.40, 95% CI = 0.17–0.97, *P* = 0.043), and high A2 DS2 score (aOR = 6.71, 95% CI = 3.10–14.52, *P* < 0.001) remained independent predictors of SAP (Table 2).

**Table 2:** Multivariable analysis of possible predictors of stroke associated pneumonia

	Crude OR	*P* value	Adjusted OR	*P* value
Hyperlipidemia	0.41 [0.20–0.87]	0.020	0.40 [0.17–0.97]	0.043
High A2DS2 score (5–10)	7.68 [3.81–15.50]	<0.001	6.71 [3.10–14.52]	<0.001
Severe leukoaraiosis	4.90 [2.42–9.93]	<0.001	4.41 [2.04–9.55]	<0.001

We used binary logistic regression adjusted for hyperlipidemia, A2DS2 score, and severe leukoaraiosis

We further analyzed characteristics according to the severity of LA in using subgroup analysis. Older age, female sex, and impaired consciousness were correlated with severe LA (Table 3). In the analysis of discharge outcomes between two groups, patients in the SAP group showed longer hospitalization duration, severer discharge NIHSS score, more frequent in-hospital mortality and intubation events (Table 4).

**Table 3:** Baseline characteristics between severe and mild LA patients

	Mild LA (*n* = 205)	Severe LA (*n* = 86)	*P* value
SAP (%)	15 (7)	24 (28)	<0.001
Age, y	62 ± 13	74 ± 10	<0.001
Sex, male (%)	138 (67)	47 (55)	0.040
Hyperlipidemia (%)	85 (41)	31 (36)	0.389
Atrial fibrillation (%)	35 (17)	18 (21)	0.437
Initial NIHSS [IQR]	6 [1–6]	6 [2–7]	0.219
Impaired consciousness (%)	13 (6)	12 (14)	0.034
Dysphagia (%)	31 (16)	22 (26)	0.062

*SAP* stroke-associated pneumonia, *LA* leukoaraiosis, *NIHSS* National Institute of Health Stroke Scale

**Table 4:** Discharge outcomes between with or without SAP

	Non-SAP (*n* = 264)	SAP (*n* = 44)	*P* value
Hospitalization duration, d [IQR]	9 [7–14]	30 [14–51]	<0.001
Discharge NIHSS score [IQR]	2 [0–4]	9 [5–18]	<0.001
In-hospital mortality, %	0 (0)	3 (7)	0.003
Event of intubation, %	1 (0)	7 (16)	<0.001

*SAP* stroke-associated pneumonia, *NIHSS* National Institute of Health Stroke Scale

## Discussion

In this study, SAP occurred in 14% of patients with acute ischemic stroke, which is similar to what has been reported in previous studies [1, 2, 17]. We also found that severe LA was independently associated with SAP in patients with acute ischemic stroke.

We graded LA in both the periventricular and subcortical areas, as the pathology is different between the two regions [18]. Our subgroup analysis according to the locations of the LA consistently revealed severe LA, which defined as 2 or more Fazekas score in each area, as a potent predictor of SAP in both the periventricular (aOR = 4.18, 95% CI = 1.94–8.99, *P* < 0.001) and subcortical areas (aOR = 3.59, 95% CI = 1.67–7.70, *P* = 0.001). Thus, the severity or burden of LA appeared to be more important rather than the location in SAP.

The association between SAP and LA may be explained by several hypotheses: First, dysphagia, which is common in patients with acute stroke (up to 67%) [5], could lead to aspiration and subsequently SAP. Patients with LA are known to be more prone to developing dysphagia according to its severity [19]. It could be caused by a disruption in the connection of white matter and reduced input to the brainstem swallowing center, leading to pseudobulbar palsy [20]. Additionally, LA has been shown to be an independent predictor of dysphagia after acute stroke [8]. Second, it is possible that LA reduces the cough reflex. Decreased dopamine production by massive structural disruption of the LA leads to reduce expression of substance P in the glossopharyngeal nerve and the cervical parasympathetic ganglion, inhibiting the initiation of the cough reflex from pharyngeal, laryngeal and tracheal epithelia, which may lead to aspiration [21]. Third, impaired cognition or consciousness caused by severe LA also increases the chance for aspiration and could have a role in SAP. In this study, we found that patients with severe LA more frequently exhibited impaired consciousness.

The strengths of our study were as following: First, it is the first study about association between SAP and LA as a radiological predictor. Second, we also confirmed that SAP is correlated with poor outcomes in the aspects of hospitalization duration, neurological function and mortality, continued to previous study [2, 3]. We also have several limitations. First, it was a single-center study with a lower statistical power. We are also cautious in generalizing the results because treatment modalities or preventive strategies for pneumonia may be different among centers. Second, we did not measure the size of the infarct, which may be important for SAP, as has been shown in other studies [2, 17, 22]. Instead, we adjusted for the NIHSS score, which is known to be well correlated with the size of the infarct. Thus, we believe that not measuring infarct size may not affect the major outcomes of this study. Third, the effects of pre-stroke functional status (e.g. modified Rankin score, cognitive status) should be considered. Fourth, we did not separate probable and definite SAP according to radiological findings. Thus, SAP group may have possibility of heterogeneous traits with different burden of SAP. Last, the type of pneumonia and its nature (e.g. aspiration pneumonia, microaspiration pneumonia) should be confounded.

## Conclusions

In conclusion, severe LA may predict SAP in patients with acute ischemic stroke. In clinical practice, careful observation of these high risk patients can be helpful to find SAP. Although these findings may be interpreted as potential hypothesis generation, further validation by larger prospective studies may be needed.

## Additional files

Additional file 1: Diagnostic criteria for stroke associated pneumonia based on the CDC criteria. (DOCX 25 kb)
Additional file 2: Calculation of A2DS2 score. (DOC 25 kb)

## Abbreviations

CMBs: Cerebral microbleeds
DWI: Diffusion weighted image
LA: Leukoaraiosis
NIHSS: the National Institutes of Health Stroke Scale
SAP: Stroke associated pneumonia
TE: Echo time
TOF: Time of flight
TR: Repetition time
