# Supplementary material for: Leukoaraiosis is associated with pneumonia after acute ischemic stroke
Source: BMC Neurol. 2017 Mar 16;17:51. doi: 10.1186/s12883-017-0830-5 (PMC5356415; doi:10.1186/s12883-017-0830-5)
Supplement: Additional file 1: — Diagnostic criteria for stroke associated pneumonia based on the CDC criteria. (DOCX 25 kb) [file 12883_2017_830_MOESM1_ESM.docx]

**Additional file 1 Diagnostic criteria for stroke associated pneumonia based on the CDC criteria**

1. At least 1 of the following
   1. Fever > 38℃ with no other recognized cause
   2. Leukopenia < 4000 WBC/mm^3^ or leukocytosis > 12000 WBC/mm^3^
   3. Altered mentality without other recognized cause for adults ≥ 70 years old
2. And at least 2 of the following
   1. New onset purulent sputum, or change in character of sputum over a 24 h period, or increased respiratory secretions, or increased suctioning requirements
   2. New onset or worsening cough, dyspnea, or tachypnea
   3. Rales, crackles, or bronchial breath sounds
   4. Worsening gas exchange
3. And ≥ 2 serial chest radiographs with at least 1 of the following
   1. New or progressive and persistent character
   2. Infiltration, consolidation, or cavitation
