# Supplementary material for: Leukoaraiosis is associated with pneumonia after acute ischemic stroke
Source: BMC Neurol. 2017 Mar 16;17:51. doi: 10.1186/s12883-017-0830-5 (PMC5356415; doi:10.1186/s12883-017-0830-5)
Supplement: Additional file 2: — Calculation of A2DS2 score. (DOC 25 kb) [file 12883_2017_830_MOESM2_ESM.doc]

**Additional file 2 Calculation of A2DS2 score**

1. **Age** ≥ 75 years = 1
2. **Atrial fibrillation** = 1
3. **Male sex** = 1
4. **Dysphagia** = 2
5. **Stroke severity**
   1. NIHSS 0-4 = 0
   2. NIHSS 5-15 = 3
   3. NIHSS ≥ 16 =5

**Total score ranges: 0 - 10**
